# Mesenchymal Stem Cells Modulate Regulatory T Cells: From Molecular Mechanisms to Disease Therapy

**DOI:** 10.1155/sci/6457805

**Published:** 2026-08-09

**Authors:** Meng Yuan, Ting Sun, Yaru Qiao, Peter Muro, Bo Wang, Xiangjun Zhou, Fei Mao

**Affiliations:** ^1^ Department of Laboratory Medicine, School of Medicine, Jiangsu University, Zhenjiang 212013, Jiangsu, China, ujs.edu.cn; ^2^ Department of Laboratory Medicine, The Affiliated People’s Hospital, Jiangsu University, Zhenjiang 212002, Jiangsu, China, ujs.edu.cn; ^3^ The People’s Hospital of Danyang, Affiliated Danyang Hospital of Nantong University, Zhenjiang 212300, Jiangsu, China; ^4^ Danyang Maternal and Child Health Hospital, Zhenjiang 212300, Jiangsu, China

## Abstract

Mesenchymal stem cells (MSCs) and regulatory T cells (Tregs) form a key immunoregulatory axis essential for maintaining immune homeostasis and treating autoimmune diseases, transplant rejection, and inflammatory disorders. Although both MSCs and Tregs have been individually studied, a clear synthesis of how MSCs regulate Treg function across distinct mechanistic layers and disease contexts remains lacking. MSCs regulate Treg differentiation, expansion, and functional stability through paracrine signaling, intercellular contact–dependent pathways, extracellular vesicle (EV)–mediated transfer of microRNAs (miRNAs) and proteins, mitochondrial transfer, and metabolic and epigenetic reprogramming. These mechanisms restore Th17/Treg balance, suppress inflammation, and promote tissue repair. Preclinical studies demonstrate strong therapeutic potential in multiple immune–mediated diseases; however, clinical translation remains limited. Unlike previous studies, this review integrates soluble and exosomal signaling pathways, compares shared and disease‐specific regulatory mechanisms, and critically evaluates MSC source variability, methodological limitations, and causes of clinical inconsistency. It aims to provide a conceptual framework to guide future mechanistic studies and clinical development of MSC–Treg–based therapies.

## 1. Introduction

Maintaining immune homeostasis relies on precise regulation of the immune system, whereas its dysregulation can lead to severe pathological conditions, such as autoimmune diseases, excessive inflammation, and transplant rejection [1, 2]. In recent years, therapeutic strategies based on cell‐mediated immune modulation have emerged as a promising approach for the treatment of these disorders. Mesenchymal stem cells (MSCs) not only possess multipotent differentiation capacity but also exhibit potent immunomodulatory properties. As stromal cells, MSCs can dynamically respond to inflammatory signals and actively regulate the immune microenvironment, thereby exerting therapeutic effects in various diseases [3].

Regulatory T cells (Tregs), which play a central role in maintaining immune tolerance, primarily restore immune balance through their expansion and functional activation [4]. Therefore, elucidating the molecular pathways by which MSCs regulate Tregs to exert therapeutic effects is critical for understanding their immunomodulatory functions. Accumulating evidence suggests a close and dynamic interaction between MSCs and Tregs [5]. MSC‐mediated regulation of Tregs involves multiple interconnected processes, including the secretion of soluble immunomodulatory factors, the release of extracellular vesicles (EVs) and exosomes, direct cell–cell interactions, and metabolic and mitochondrial regulation [6–9]. These mechanisms collectively influence Treg differentiation, stability, and suppressive activity. In addition, emerging studies suggest that epigenetic regulation and intracellular signaling pathways further contribute to shaping the MSC–Treg regulatory axis [8, 10]. Together, these processes form a complex regulatory network that is essential for maintaining immune tolerance under both physiological and pathological conditions.

Despite these advances, the regulatory network linking MSCs and Tregs remains highly complex, and current evidence is still fragmented across different experimental models. A comprehensive synthesis of these findings is therefore necessary to clarify the underlying mechanisms and their therapeutic implications. This review aims to address these gaps by systematically integrating mechanistic, disease‐specific, and translational perspectives. We first summarize the diverse molecular pathways through which MSCs modulate Tregs, with particular emphasis on a comparative analysis of soluble factor‐ and exosome‐mediated regulation. We then examine the role of the MSC–Treg axis across autoimmune diseases, transplantation, and inflammatory disorders, highlighting both common and disease‐specific mechanisms as well as the key determinants of heterogeneous therapeutic outcomes. Finally, we critically discuss recent clinical failures and methodological limitations, including variability in MSC sources and culture conditions, the lack of standardized potency assays, and Treg instability, which have hindered clinical translation. By bridging mechanistic insights with translational realities, this review provides a conceptual framework to guide future research and accelerate the development of MSC–Treg–based therapies.

## 2. Biological Basis of MSC–Treg Interaction

### 2.1. Definition and Functional Characteristics of MSCs

MSCs are multipotent mesenchymal stromal cells widely distributed throughout various tissues of the human body and characterized by strong in vitro proliferative capacity [11]. According to the criteria established by the International Society for Cell and Gene Therapy (ISCT), MSCs are defined by their plastic adherence under standard culture conditions; expression of surface markers CD73, CD90, and CD105; absence of hematopoietic and endothelial markers (CD11b, CD14, CD19, CD34, CD45, CD79a, and human leukocyte antigen [HLA]‐DR); and their capacity to differentiate in vitro into adipocytes, chondrocytes, and osteoblasts [12]. MSCs have been identified in multiple tissue sources, including bone marrow, adipose tissue, umbilical cord, dental pulp, and placenta, and MSCs derived from different tissues may exhibit variations in biological characteristics and clinical applications [13]. These cells possess multipotent differentiation potential and self‐renewal capacity, enabling them to differentiate into functional cell types of various tissues and organs under appropriate induction conditions [14]. The fundamental biological properties of MSCs include plastic adherence, clonogenicity, and multilineage differentiation potential [15]. In addition, MSCs exhibit low immunogenicity, tissue‐homing capacity, and potent immunomodulatory functions [16]. Among these characteristics, immunomodulation plays a particularly important role in MSC‐based therapies for immune‐related diseases such as inflammatory bowel disease (IBD) [17]. This immunomodulatory activity is primarily mediated by direct cell–cell interactions with immune cells and by paracrine secretion of various soluble factors [18]. Owing to these multifunctional properties, MSCs demonstrate considerable therapeutic potential in the treatment of immune‐mediated and inflammatory diseases.

### 2.2. Treg Subsets and Functional Plasticity

Tregs can be classified into several subsets based on their developmental origin and functional characteristics, including thymus‐derived Tregs (tTregs), peripherally induced Tregs (pTregs), and in vitro–induced Tregs (iTregs). Among these, pTregs and iTregs are collectively referred to as induced iTregs [19]. Tregs primarily originate from the thymic medulla and represent the dominant Treg population [20]. Their formation depends on high‐affinity recognition of self‐antigens by the T cell receptor (TCR) during thymic selection. In contrast, iTregs differentiate from thymus‐independent, nonsensitized CD4^+^CD25^−^Foxp3^−^ T cells (naïve T cells) following TCR activation, under the combined influence of transforming growth factor‐β (TGF‐β) and interleukin‐2 (IL‐2) [21, 22].

Treg plasticity refers to the ability of Tregs to dynamically adapt to different immune microenvironments through phenotypic reprogramming and functional modulation, thereby maintaining immune homeostasis [23]. Following the discovery of the transcription factor forkhead box P3 (Foxp3), Shimon Sakaguchi demonstrated through a series of studies in 2003 that Foxp3 is the core transcription factor regulating and maintaining Treg development and functional stability [24]. Although Foxp3 plays a central role in Treg biology, its expression alone is insufficient to regulate the entire Treg transcriptional program. For example, activated conventional human T cells can transiently express Foxp3 without acquiring an immunosuppressive function. Genome‐wide chromatin immunoprecipitation sequencing (ChIP‐seq) analysis has shown that only a limited proportion of genes that require Foxp3 expression directly bind Foxp3, suggesting that most transcriptional regulation in Tregs involves additional transcription factors acting independently or cooperatively with Foxp3 [25].

Helios is one such transcription factor involved in the regulation of Treg transcriptional activity. As a member of the Ikaros family of transcription factors, Helios is expressed in Foxp3^+^ T cells and contributes to maintaining Treg immunosuppressive function and phenotypic stability [26]. Helios regulates the STAT5 signaling pathway, thereby supporting sustained Foxp3 expression and enabling Tregs to retain their suppressive function under inflammatory conditions [27]. However, subsequent studies have indicated that while Helios contributes to Treg stability in mouse models, in humans, it primarily serves as a marker of Treg phenotypic and functional stability rather than as a driver of stability [28]. In addition to transcriptional regulation, metabolic programming plays a crucial role in Treg functional plasticity. To meet their energetic demands, Tregs dynamically regulate several metabolic pathways, including glycolysis, oxidative phosphorylation (OXPHOS), fatty acid oxidation (FAO), and amino acid metabolism [29]. Under physiological conditions, Tregs predominantly rely on FAO and have a relatively low glycolytic activity to maintain their immunosuppressive function. In contrast, Foxp3‐deficient Tregs undergo metabolic reprogramming via activation of the mTORC2‐Foxo1 signaling axis, shifting towards an effector T cell (Teff)–like metabolic phenotype characterized by elevated glycolysis and altered OXPHOS, ultimately leading to functional impairment. Targeting metabolic pathways such as mTORC2 signaling and glycolysis to restore Treg function has therefore emerged as a promising therapeutic strategy for immune‐related diseases [30]. Overall, the functional plasticity and stability of Treg cells are regulated through the coordinated control of key transcription factors, including Foxp3 and Helios, together with distinct metabolic programs.

## 3. Mechanisms of MSC‐Mediated Regulation of Treg Cells

Accumulating evidence indicates that MSCs regulate immune homeostasis through multiple coordinated mechanisms, with a central role in promoting the induction, expansion, and functional stabilization of Tregs. These regulatory effects are primarily mediated by the secretion of soluble immunomodulatory factors and by direct cell–cell contact. The principal mechanisms by which MSCs regulate Treg cells are discussed below. Understanding these mechanisms provides an important theoretical basis for the development of novel immunotherapeutic approaches.

### 3.1. Regulation via Soluble Immunomodulatory Factors

Upon activation, MSCs secrete a variety of soluble mediators with potent immunomodulatory activity. These molecules form a complex regulatory network within the local microenvironment, directly or indirectly guiding the differentiation of naïve T cells into Tregs while enhancing their suppressive capacity [31]. TGF‐β is a key cytokine driving the differentiation of naïve CD4^+^ T cells into Tregs. Studies indicate that TGF‐β secreted by MSCs can restore the Treg/Th17 balance, thereby protecting tissues from injury in an acute respiratory distress syndrome (ARDS) cell model [32]. Similar regulatory effects have been confirmed in mouse models of colon cancer [33]. In experimental colitis, intraperitoneally injected MSCs migrate to mesenteric lymph nodes, where they secrete TGF‐β and promote local Treg differentiation while reducing Th17 cells, thereby alleviating intestinal inflammation [34].

Indoleamine 2,3‐dioxygenase (IDO) represents another critical mediator of MSC immunoregulation [35]. IDO suppresses Teff responses and promotes Treg differentiation by depleting tryptophan and accumulating kynurenine in the microenvironment [36]. Inflammatory stimuli, such as interferon‐γ (IFN‐γ), can markedly enhance IDO expression in MSCs through pathways including AMPK‐mTOR signaling, thereby strengthening Treg induction in acute inflammatory models, such as liver injury [37]. In cardiomyopathy models, repeated MSC infusion upregulates IDO expression, promotes Treg differentiation, enhances Th2 responses, suppresses Th1/Th17 activity, and reduces myocardial inflammation [38]. However, in the tumor microenvironment of acute myeloid leukemia (AML), IFN‐γ released by leukemic cells induces high IDO expression in MSCs, promoting Treg expansion and establishing an immunosuppressive microenvironment that facilitates leukemia progression [39]. This finding reveals the dual positive and negative effects, MSCs may exert under pathological conditions.

**Figure 1:**
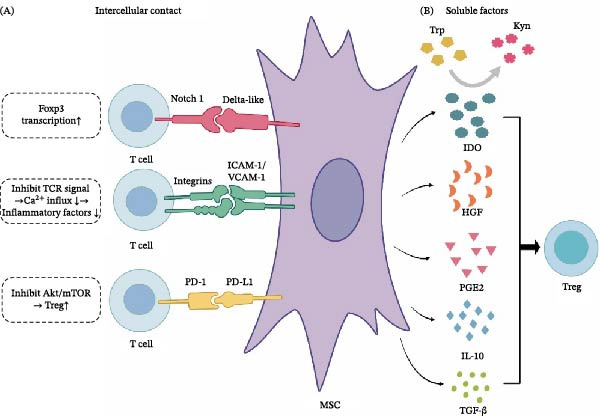
Schematic illustration of MSC‐mediated Treg regulation via soluble factors and cell contact. (A) Contact‐dependent pathways, including Notch signaling, ICAM‐1/VCAM‐1 interactions, and PD‐L1/PD‐1 signaling. (B) Soluble factors secreted by MSCs, such as IDO, HGF, PGE2, IL‐10, and TGF‐β.

Although interleukin‐10 (IL‐10) does not directly induce Treg polarization, it plays an essential role in maintaining Treg suppressive function [40, 41]. Blocking IL‐10 weakens Treg‐mediated immunosuppression [42]. Furthermore, studies have revealed that EVs derived from IL‐10–overexpressing MSCs can upregulate Treg differentiation while suppressing Th1/Th17 cells [43]. MSCs also regulate T cell differentiation by secreting prostaglandin E2 (PGE2), which acts via the EP4 receptor on T cells to inhibit Th17 differentiation and reduce IL‐17A production [44]. Additionally, PGE2 can reprogram differentiated Th17 cells into regulatory‐like cells, indicating its role in MSC‐mediated Treg phenotypic conversion [45]. Hepatocyte growth factor (HGF) secreted by MSCs further contributes to the conversion of differentiated Th17 cells into functional Tregs [46]. Together, these findings indicate that MSCs promote Treg induction and expansion through the synergistic actions of multiple soluble mediators, as summarized in Table 1 and illustrated in Figure 1.

**Table 1:** Major soluble factors secreted by MSCs and their regulatory effects on Treg cells.

The type of soluble factors	Sources of MSCs	Mechanism of action	Effects of MSCs on Tregs	References
TGF‐β	BM‐MSCs hUC‐MSCs	Suppress miR‐155 expression and stabilize Foxp3; local secretion at target organs (such as mesenteric lymph nodes)	Inducing CD4^+^ T cells to differentiate into Tregs; restore Treg/Th17 balance	[32–34]
IDO	Multiple types of MSCs	Consumes tryptophan, accumulates kynurenine; regulated by inflammatory signals such as IFN‐γ, its expression is upregulated via the AMPK‐mTOR pathway	Promotes Treg differentiation; suppresses effector T cells; bidirectional effects (tumor‐promoting in AML)	[36–39]
IL‐10	IL‐10–overexpressing MSCs	As a Treg effector molecule, it maintains Foxp3 stability	Maintaining Treg function (nonpolarizing induction); enhance Treg differentiation and suppress Th1/Th17 cell activity; blocking IL‐10 weakens Treg function	[42, 43]
PGE2	BM‐MSCs	Signaling through the EP4 receptor; inhibiting Th17 differentiation; reprogramming Th17 cells to a regulatory phenotype	Inhibits IL‐17A secretion and Th17 cell generation via EP4 receptor; induces Treg phenotypic conversion; enhances Treg suppressive function	[44, 45]
HGF	BM‐MSCs	Induce Th17‐to‐Treg conversion; regulate the Treg/Th17 cell balance	Promoting the conversion of fully differentiated Th17 cells into functional Treg cells	[46]

Abbreviations: AML, acute myeloid leukemia; BM‐MSCs, bone marrow–derived mesenchymal stem cells; HGF, hepatocyte growth factor; hUC‐MSCs, human umbilical cord–derived mesenchymal stem cells; IDO, indoleamine 2,3‐dioxygenase; IL‐10, interleukin‐10; MSCs, mesenchymal stem cells; PGE2, prostaglandin E2; TGF‐β, transforming growth factor‐β; Th, T helper cell; Treg, regulatory T cell.

### 3.2. Cell‐Contact–Dependent Regulatory Mechanisms

#### 3.2.1. Programmed Death‐Ligand 1 (PD‐L1)/PD‐1 Signaling

Direct cell–cell interactions represent an important mechanism by which MSCs regulate Treg responses. MSCs express high levels of PD‐L1, which interacts with PD‐1 on activated T cells to transmit inhibitory signals [47, 48]. This interaction promotes T cell inactivation, apoptosis, or differentiation into Tregs. Mechanistically, the process involves the inhibition of the Akt/mTOR pathway [49] and may also involve cytokines such as IL‐10 [50]. The PD‐1/PD‐L1 pathway not only promotes Treg differentiation but also suppresses the proliferation of Teffs such as Th1 and Th17, thereby synergistically enhancing immunosuppressive effects [51]. For example, tonsil‐derived MSCs directly inhibit STAT3 phosphorylation in CD4^+^ T cells via the PD‐L1/PD‐1 axis, thereby reducing Th17 cell generation in vivo [52].

In tumor microenvironments, MSCs can further upregulate PD‐L1 expression and synergize with IDO to promote Treg expansion, thereby suppressing CAR‐T cell function [53]. These findings suggest that modulation of the PD‐L1 expression on MSCs may represent a strategy to optimize immunotherapeutic efficacy.

#### 3.2.2. Adhesion Molecules: Intercellular Adhesion Molecule‐1 (ICAM‐1) and Vascular Cell Adhesion Molecule‐1 (VCAM‐1)

Inflammatory cytokines significantly enhance the expression of ICAM‐1 and VCAM‐1 on MSCs, strengthening their adhesion capacity. These molecules are essential mediators of cell recognition and interaction, contributing to MSC‐mediated immunoregulation [54]. ICAM‐1 on MSCs can bind to CD43 on activated T cells, disrupting TCR microcluster formation and rapidly inhibiting downstream calcium signaling and proinflammatory cytokine transcription [55]. Through this interaction between ICAM‐1 and CD43, MSCs rapidly suppress T cell activation and help control acute inflammatory responses. In IBD models, engineered MSCs overexpressing ICAM‐1 significantly increased the proportion of Tregs in the spleen compared to conventional MSCs while exhibiting stronger suppression of Th1 and Th17 cells [56]. Similarly, VCAM‐1–overexpressing MSCs promote Treg expansion and suppress Th1/Th17 responses in colitis models [57]. Moreover, VCAM‐1 serves as a marker for identifying MSC subpopulations with unique immunomodulatory activity. Heterogeneous VCAM‐1 expression has been observed among MSCs from different sources. For instance, CD106^+^ chorionic villus–derived MSCs display stronger immunomodulatory capacity [58]. These findings suggest that VCAM‐1 may serve both as a functional molecule and as a marker for selecting therapeutically superior MSC populations.

#### 3.2.3. Notch Signaling Pathway

The Notch signaling pathway is a highly conserved system that regulates cell–cell communication and gene transcription [59]. It has been documented that the Notch1 receptor on T cells plays a crucial role in MSC‐induced Treg differentiation. Upon activation, Notch1 directly binds to the Foxp3 promoter, promoting Foxp3 transcription and driving Treg development [60]. When MSCs detect tissue injury or pathogen‐associated signals through Toll‐like receptors 3 or 4, the expression of the Notch ligand Delta‐like 1 on their surface is significantly increased. This upregulation enhances MSCs’ capacity to induce Tregs. Inhibition of the Notch pathway abolishes this effect, confirming that Notch signaling represents a critical intermediate step in MSC‐mediated Treg induction [10]. These findings indicate that Notch signaling not only mediates MSC‐driven Treg differentiation but is also finely regulated by microenvironmental cues.

### 3.3. MSC‐Derived Exosomes and EVs

MSC‐derived exosomes and EVs have emerged as key mediators of MSC immunomodulatory activity. These membrane‐bound vesicles contain proteins, nucleic acids, and other bioactive molecules that regulate immune responses [61, 62].

MicroRNAs (miRNAs) represent important functional components of MSC‐EVs. Numerous studies demonstrate that specific miRNAs play critical roles in EV‐mediated Treg regulation [63]. For example, exosomes from 3D‐cultured MSCs restore the Th17/Treg balance in periodontitis and experimental colitis by delivering miR‐1246, which suppresses Nfat5 expression [64]. Similarly, umbilical cord blood MSC–derived vesicles promote M2 macrophage polarization and Treg generation through miR‐100‐5p in autoimmune scleritis models [65]. Other studies show that MSC‐EV–derived miRNAs, such as let‐7f‐5p, regulate the RORC/IL‐17A axis to correct immune imbalance in Sjögren’s syndrome [66]. In transplantation models, MSC‐EVs promote Treg differentiation via the miR‐638/Fosb axis, thereby prolonging graft survival [67]. Beyond miRNAs, long noncoding RNAs (lncRNAs) delivered by MSC‐derived exosomes can also promote Treg differentiation by downregulating proteins such as Sirtuin 1 (SIRT1), a NAD^+^‐dependent deacetylase [68]. In addition to nucleic acids, MSC‐EVs contain functional proteins that can directly activate signaling pathways in recipient cells. For example, in systemic lupus erythematosus (SLE) models, umbilical cord MSC–derived exosomes deliver Foxp1 to stabilize Foxp3 transcription and promote Treg induction, alleviating lupus nephritis [69]. Moreover, MSC‐derived exosomes can activate STAT5 signaling and the autophagy pathways in Tregs, thereby further enhancing the Foxp3 expression and immunoregulatory capacity [70].

### 3.4. Metabolic and Epigenetic Regulation of Tregs

#### 3.4.1. Mitochondrial Transfer

A major challenge in Treg‐based therapies is the instability of Tregs in inflammatory environments, where the Foxp3 expression may be lost. It has been documented that human bone marrow–derived MSCs (BM‐MSCs) can transfer mitochondria to induced Tregs via tunneling nanotubes, thereby enhancing Foxp3 stability through regulators such as BACH2 and SENP3 [71]. This finding provides a mechanistic explanation for how MSCs enable Tregs to maintain function under inflammatory conditions [71]. This mechanism explains how MSCs enhance Treg resistance to inflammatory stress. Early studies conducted nearly two decades ago had already shown that MSCs could restore cellular function in mitochondria‐deficient cells by transferring healthy mitochondria [72]. Subsequent investigations revealed that mitochondrial transfer is an important mechanism by which MSCs promote Treg differentiation and suppress inflammatory responses [9]. Mechanistically, mitochondrial transfer from MSCs to allogeneic Tregs has been shown to be dependent on HLA compatibility, with transfer efficiency correlating with the degree of mismatch in donor‐recipient HLA‐C and HLA‐DRB1 epitopes. After acquiring mitochondria, Tregs exhibit enhanced adenosine production, thereby strengthening their immunosuppressive activity [73]. Importantly, mitochondrial transfer by MSCs is not limited to natural Tregs but also affects genetically engineered T cells. Evidence suggests that MSCs can induce activated CD3^+^ T cells to reprogram toward a Treg‐like phenotype via mitochondrial transfer while simultaneously improving the survival of multiple T cell subsets [74]. This finding highlights a potential strategy to mitigate T cell exhaustion in chimeric antigen receptor T‐cell therapies. The relevance of mitochondrial transfer has also been demonstrated in acute inflammatory diseases. In an experimental ischemic stroke model, umbilical cord–derived MSCs restored the Th17/Treg balance by transferring mitochondria to CD4^+^ T cells, thereby reversing stroke‐induced T cell dysregulation and alleviating neuroinflammation [75].

Collectively, these findings suggest that mitochondrial transfer represents an important mechanism through which MSCs regulate Treg function and restore immune homeostasis, with potential therapeutic implications for both chronic immune disorders and acute inflammatory conditions.

#### 3.4.2. Lactate Metabolism and FAO

In the tumor microenvironment or inflammatory sites, when glucose is extensively consumed by Teffs and tumor cells, Tregs can switch to utilizing lactate to maintain their function [76]. Depending on the microenvironment, MSCs can either enhance or suppress Treg function via lactate metabolism. For instance, MSC‐derived exosomes can deliver miR‐124, which targets and inhibits the Treg lactate transporter MCT1, thereby reducing lactate uptake. This process weakens Treg‐mediated immunosuppression and enhances antitumor immune responses [77]. Conversely, under inflammatory conditions, MSCs undergo metabolic reprogramming characterized by increased glycolysis and elevated lactate production. Lactate released from MSCs has been shown to directly inhibit the differentiation of CD4^+^ T cells into proinflammatory Th1 and Th17 subsets. When lactate production in MSCs is pharmacologically inhibited, their overall immunosuppressive capacity is markedly reduced [78].

FAO represents a central metabolic pathway that maintains Treg stability and immunosuppressive function, distinguishing Tregs metabolically from proinflammatory T cells [79, 80]. Peroxisome proliferator–activated receptors (PPARβ/δ) regulate FAO and glucose uptake, and these metabolic processes contribute to MSC‐mediated regulation of Th17/Treg balance in vitro [81]. However, direct evidence demonstrating the MSC regulation of FAO in Tregs remains limited. It is therefore plausible that MSCs may influence Treg FAO through several mechanisms, including the provision of metabolic substrates, the secretion of bioactive mediators that regulate key metabolic enzymes, or mitochondrial transfer. Further investigation is required to clarify these potential regulatory pathways.

#### 3.4.3. Epigenetic Regulation of Foxp3

**Figure 2:**
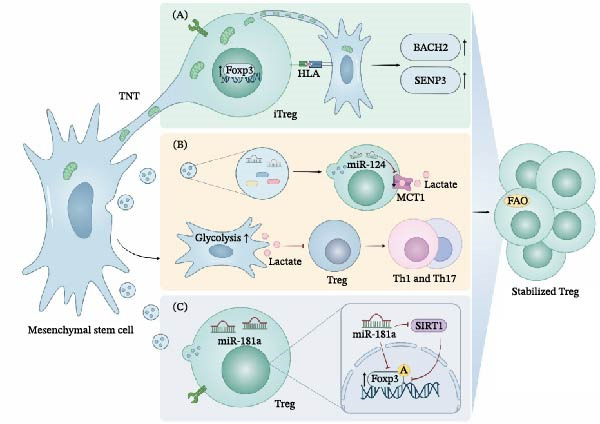
Metabolic and epigenetic regulation of Tregs by MSCs. (A) Mitochondrial transfer: Mitochondrial transfer via tunneling nanotubes stabilizes Foxp3 expression. (B) Metabolic reprogramming: In the tumor microenvironment, exosomal miR‐124 inhibits lactate uptake in Tregs, while under inflammatory conditions, MSC‐derived lactate suppresses Th1/Th17 differentiation. (C) Epigenetic modulation: Exosomal miR‐181a enhances Foxp3 acetylation via SIRT1 inhibition.

The lineage stability and functional specialization of Tregs are tightly controlled by epigenetic mechanisms, including regulatory elements at the Foxp3 locus, DNA demethylation, and histone modifications [82]. As previously mentioned, MSC‐derived TGF‐β can stabilize Foxp3 expression by suppressing miR‐155, representing a form of posttranscriptional epigenetic regulation [32]. In addition, studies have shown that MSC‐derived exosomes deliver specific miRNAs, such as miR‐181a, which enhance Foxp3 acetylation by inhibiting the deacetylase SIRT1 in recipient cells. This posttranslational modification increases Foxp3 stability and transcriptional activity, thereby reinforcing the Treg suppressive function [83]. Furthermore, coculture systems used to expand Tregs in the presence of MSCs have revealed a significant increase in the proportion of Foxp3^+^/Helios^+^ Tregs, suggesting that MSCs may create a microenvironment that maintains a stable epigenetic program in Tregs [84]. Together, these metabolic and epigenetic mechanisms through which MSCs regulate Treg stability and function are summarized in Figure 2.

### 3.5. Comparison Between Soluble Factor–Mediated and Exosome‐Mediated Pathways

Although the above mechanisms collectively mediate MSC regulation of Tregs, their relative contributions likely differ substantially across pathological microenvironments. In the early phase of acute inflammation, such as in acute colitis or liver injury, rapidly secreted soluble factors (e.g., TGF‐β, PGE2, and IDO‐derived kynurenine) likely play the predominant role [34, 37]. These molecules can act within minutes to hours, directly engaging TCRs or metabolic pathways to restrain excessive inflammation. In contrast, exosome‐mediated regulation may become more important in chronic inflammatory settings or when long‐term Treg stability is required [85]. Unlike single‐target regulation of cytokines, MSC‐EVs can simultaneously deliver multiple functional components (miRNAs, lncRNAs, and functional proteins) to Tregs, thereby achieving multilevel regulation from epigenetic modification to metabolic reprogramming [66, 69].

It should be emphasized that these two pathways are not mutually exclusive but exhibit significant synergistic effects. Evidence indicates that IFN‐γ priming simultaneously upregulates IDO expression and enriches immunomodulatory miRNAs (e.g., miR‐125a and miR‐21) within MSC‐derived exosomes, suggesting a coordinated, synergistic program [37, 86].

Notably, some studies have demonstrated that the effects of MSC‐EVs on Treg induction are independent of soluble factors [70], whereas other studies suggest that these two components are experimentally difficult to completely dissociate [87], which directly confounds the assessment of the true biological functions of EVs. This represents a core issue that must be addressed in future research.

## 4. MSC–Treg Axis in Disease Pathophysiology

The immunoregulatory relationship between MSCs and Tregs operates within complex microenvironments of the disease. Across various pathological conditions, this axis exerts immunosuppressive effects through distinct molecular pathways, as described in the preceding section. The current discussion focuses on the specific roles of the MSC–Treg axis in diverse disease settings.

### 4.1. Autoimmune Diseases

#### 4.1.1. IBD

**Figure 3:**
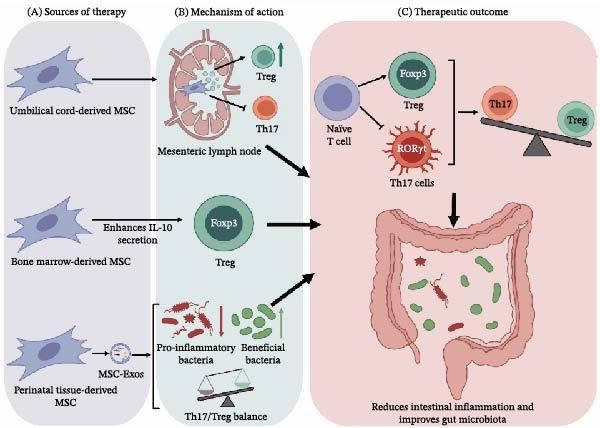
Therapeutic mechanisms of MSCs and MSC‐derived exosomes in IBD. (A) Sources of MSCs and exosomes. (B) Mechanisms of action: UC‐MSCs home to mesenteric lymph nodes and secrete TGF‐β1; BM‐MSCs enhance Treg function and IL‐10 secretion; perinatal MSC–exosomes modulate Treg/Th17 balance and gut microbiota. (C) Therapeutic outcomes: restored intestinal immune balance and reduced inflammation.

Evidence from multiple studies demonstrates that MSCs from different sources and their exosomes effectively ameliorate DSS‐induced IBD (Figure 3). Exosomes derived from adipose tissue MSCs can directly induce Treg differentiation and suppress proinflammatory cytokines, thereby reducing acute inflammation [88]. Umbilical cord–derived MSCs localize to mesenteric lymph nodes, where they secrete TGF‐β1 to enhance Treg generation and suppress Th17 cells, illustrating targeted immunomodulation within lymphoid tissues [34].

BM‐MSCs alleviate IBD by promoting the immunosuppressive activity of CD4^+^CD25^+^Foxp3^+^ Tregs and stimulating the secretion of anti‐inflammatory cytokines [89]. Similarly, perinatal tissue–derived MSC exosomes not only modulate the Th17/Treg balance but also favorably alter gut microbiota composition, reducing proinflammatory bacterial populations and exerting dual immunoregulatory effects [90]. Collectively, these findings indicate that the MSC–Treg axis mitigates IBD through organ‐specific targeting, cellular induction, and modulation of the microbiome.

#### 4.1.2. SLE

SLE is a systemic autoimmune disorder affecting multiple organs [91]. The MSC–Treg axis contributes to SLE management by altering immune dysregulation and limiting autoantibody production. MSC transplantation restores Treg/Th17/Th1 balance through TGF‐β1 secretion and increases regulatory B10 cells, achieving comprehensive lymphocyte regulation [92]. MSC‐derived exosomes can reduce multiorgan damage by inducing spleen M2 macrophage polarization and increasing Treg populations [93]. Advanced strategies involve bioengineering MSCs or their exosomes, such as exosomes overexpressing Foxp1, which enhance Treg differentiation via the Foxp1/STAT5/Foxp3 pathway and show pronounced therapeutic potential in SLE models [69]. Additionally, MSC therapy can modulate the Th17/Treg ratio toward Treg dominance and decrease autoantibody levels [94]. Interestingly, MSC transplantation can trigger neutrophil aggregation in the bone marrow, leading to bursts of EVs that correlate with therapeutic efficacy, revealing a novel regulatory mechanism [95]. These observations underscore that the MSC–Treg axis provides both foundational immune modification and enhanced functionality in SLE through engineered modifications.

#### 4.1.3. Rheumatoid Arthritis (RA)

RA is characterized by an imbalance between Th17 and Treg cells, with inflammatory cytokines such as IL‐17 and IL‐22 accelerating disease progression [96]. Restoring this balance is essential for RA treatment. MSC‐derived exosomes exhibit potent immunosuppressive effects by increasing Treg numbers and reducing inflammation, highlighting their therapeutic potential [97]. Umbilical cord–derived MSCs upregulate Foxp3 and downregulate RORγt (retinoic acid receptor–related orphan receptor gamma t), thereby improving the Treg/Th17 ratio and alleviating arthritis symptoms in vitro and in vivo [98]. Embryonic stem cell–derived MSCs also demonstrate strong Treg‐inducing and infiltrating capabilities [99]. To overcome limitations such as short MSC survival in inflamed tissues, researchers have engineered “Janus‐faced” MSCs that combine chemotactic and immunomodulatory functions with sustained local release of free‐radical–scavenging nanoparticles, thereby achieving prolonged therapeutic efficacy in CIA mouse models [100].

### 4.2. Transplantation and Graft‐Versus‐Host Disease (GVHD)

GVHD is a severe complication following allogeneic hematopoietic stem cell transplantation caused by donor immune cell attack on recipient tissues, often resulting in high mortality [101]. MSC‐derived exosomes have emerged as a promising therapeutic option due to low immunogenicity and ease of storage [102]. Extensive studies have demonstrated MSCs’ immunomodulatory effects in mitigating GVHD [103]. MSCs can activate and expand Tregs and synergize with myeloid‐derived suppressor cells (MDSCs), offering novel avenues for GVHD prevention and treatment [104]. In solid organ transplantation, MSCs cultured in mixed lymphocyte response–conditioned medium secrete exosomes enriched in miR‐638, which promote Treg differentiation via the miR‐638/Fosb axis, enhancing graft survival [67]. Genetically modified MSCs overexpressing PD‐L1 yield small EVs that induce Tregs via the PD‐1/PD‐L1 pathway, thereby further extending allogeneic graft longevity in preclinical models [105]. Moreover, combining MSCs with immunosuppressive agents such as tocilizumab enhances splenic Treg proportions and IL‐10/Foxp3 expression, suggesting potential clinical applications in desensitization therapy [106]. These findings highlight the potential synergy of MSC–Treg–based strategies with other immunomodulatory interventions.

### 4.3. Other Inflammatory and Immune‐Mediated Disorders

Beyond autoimmune disease and transplantation, the MSC–Treg axis is pivotal in controlling inflammation and promoting tissue repair. In myocardial infarction (MI) models, pericardial injection of MSC‐derived exosomes activates Foxo3 signaling, induces Treg differentiation, and facilitates migration to damaged cardiac tissue, reducing inflammation and supporting repair [107]. In osteoarthritis (OA), MSCs modulate the Th1/Th2 and Th17/Treg balance to suppress inflammation and aid tissue regeneration [108]. MSC–Treg therapy also enhances neuroprotection following traumatic brain injury [109]. In conditions such as allergic rhinitis (AR), MSC‐derived exosomes regulate the Treg/Th17 balance via TGF‐β, Wnt, and STAT5 signaling pathways, restoring Th1/Th2 homeostasis [110]. In contrast, direct MSC–Treg activation via ICOS‐ICOSL interactions can suppress group 2 innate lymphoid cell (ILC2)–mediated inflammation [111]. These findings collectively highlight the broad immunoregulatory and therapeutic potential of the MSC–Treg axis across diverse diseases, as summarized in Table 2.

**Table 2:** The role of the MSC–Treg axis and its therapeutic effects in different diseases.

Disease category	Specific disease models	Sources of MSCs	Efficacy of Treg	Efficacy of effector T cells	Therapeutic effect	References
Autoimmune diseases	IBD	Adipose‐derived MSCs BM‐MSCs hUC‐MSCs	Treg proportion ↑ Treg function ↑ Foxp3 expression ↑	Th17 ↓ TNF‐α ↓ IL‐17 ↓	Restoration of colon length Weight regain Improved histological scores Normalization of gut microbiota	[34, 88–90]
	SLE	hUC‐MSCs	Treg proportion ↑ Treg function ↑ Foxp3 expression ↑	Th17 ↓ Th1 ↓ Autoantibodies↓	Nephritis reduced Skin damage ↓ Multiple organ damage ↓ Survival rate ↑	[69, 92–95]
	RA	Adipose‐derived MSCs hUC‐MSCs hESC‐MSCs	Treg proportion ↑ Foxp3 expression ↑ Treg infiltration ↑	Th17 ↓ IL‐17 ↓ RORγt ↓	Arthritis index ↓ Synovial proliferation ↓ Bone erosion ↓	[97–100]
Transplantation and graft‐versus‐host disease	GVHD	hUC‐MSCs BM‐MSCs	Treg proportion ↑ Treg function ↑	Effector T cells ↓ CTL activity ↓	GVHD symptoms ↓ Survival rate ↑ Target organ damage ↓	[102–104]
	Organ transplantation	BM‐MSCs	Treg proportion ↑ Treg function ↑ Foxp3 expression ↑	Effector T cells ↓ Th1/Th17 ↓ CTL activity ↓	Graft survival time ↑ Rejection response ↓ Immune tolerance established	[67, 105, 106]
Other inflammatory and immune‐mediated disorders	MI	MSC‐derived exosomes	Treg differentiation ↑ Treg migration ↑	Inflammatory cell infiltration ↓ Proinflammatory factors ↓	Improved cardiac function Reduced infarct size ↓ Reduced fibrosis ↓	[107]
	OA	Unknown MSC	Treg proportion ↑	Th1 ↓ Th17 ↓	Cartilage degeneration ↓ Inflammation ↓ Pain ↓	[108]
	AR	MSC‐derived exosomes	Treg activation ↑ Treg proportion ↑	Th2 ↓ ILC2 function ↓ IgE ↓	Allergic symptoms ↓ Nasal mucosal inflammation ↓	[110, 111]

*Note*: ↑ indicates an increase or upregulation; ↓ indicates a decrease or downregulation.

Abbreviations: AR, allergic rhinitis; BM‐MSC, bone marrow–derived mesenchymal stem cell; CTL, cytotoxic T lymphocyte; Foxp3, forkhead box P3; GVHD, graft‐versus‐host disease; hESC‐MSC, human embryonic stem cell–derived mesenchymal stem cell; hUC‐MSC, human umbilical cord–derived mesenchymal stem cell; IBD, inflammatory bowel disease; IgE, immunoglobulin E; IL, interleukin; ILC2s, group 2 innate lymphoid cells; MI, myocardial infarction; MSC, mesenchymal stem cell; OA, osteoarthritis; RA, rheumatoid arthritis; RORγt, retinoic acid receptor–related orphan receptor gamma t; SLE, systemic lupus erythematosus; Th, T helper cell; TNF‐α, tumor necrosis factor‐α; Treg, regulatory T cell.

### 4.4. Comparative and Translational Perspectives on the MSC–Treg Axis Across Diseases

#### 4.4.1. Common Mechanisms Across Diseases

In a wide range of disease models examined to date, MSCs promote Treg differentiation, expansion, and functional stabilization via the secretion of TGF‐β, IDO, and PGE2 [112, 113], as well as through exosomal delivery of immunomodulatory miRNAs [65, 83] (e.g., miR‐100‐5p and miR‐181a). A common downstream outcome is the restoration of the Th17/Treg balance, suppression of proinflammatory cytokines (IL‐17, IFN‐γ, and TNF‐α), and induction of anti‐inflammatory mediators such as IL‐10 [98, 112]. These common pathways explain why MSCs exhibit therapeutic effects in a variety of immune‐mediated diseases.

#### 4.4.2. Disease‐Specific Mechanisms

In addition to the common mechanisms described above, the MSC–Treg axis forms specific regulatory mechanisms tailored to the unique pathological features of particular diseases.

IBD involves a disrupted intestinal epithelial barrier and a dysregulated gut microbiota. MSC therapy in IBD not only induces Tregs but also promotes mucosal healing and restores microbial composition, mechanisms less relevant in systemic autoimmune diseases [90]. In SLE, a systemic autoimmune disease characterized by full‐spectrum immune dysregulation, the MSC–Treg axis exerts comprehensive immune regulation by synchronously modulating multiple immune cell compartments. MSC therapy has been shown to additionally expand regulatory B10 cells [92] and polarize macrophages toward an M2 phenotype [93], which is not required for the treatment of localized inflammatory diseases. In RA, the inflammatory joint microenvironment is dominated by Th17 cells. MSC‐derived exosomes and soluble factors such as PGE2 and HGF are particularly effective at converting pathogenic Th17 cells into Tregs [46], a mechanism that may be more critical in RA than in Th1‐driven diseases. In GVHD, the primary challenge is controlling alloreactive donor T cells while preserving protective immunity. In the context of allogeneic transplantation, the efficiency of mitochondrial transfer from MSCs to Tregs depends on HLA matching and is positively correlated with the HLA‐C and HLA‐DRB1 epitope mismatch load between the donor and recipient [9, 73]. This directly determines the level of immunosuppressive activity of Tregs and the efficacy of inducing immune tolerance to allogeneic grafts.

#### 4.4.3. Determinants of Heterogeneous Therapeutic Outcomes Across Diseases

For diseases with localized inflammatory lesions (such as IBD and OA), MSCs can achieve targeted homing to mesenteric lymph nodes or direct injection into the articular cavity, resulting in high local drug concentrations and significant efficacy [34, 114]. In contrast, for systemic multiorgan diseases (such as SLE), MSCs are difficult to penetrate the blood‐brain barrier, the glomerular basement membrane, and other tissue barriers, resulting in low target organ accumulation and poor efficacy [115].

Acute inflammatory microenvironments (such as active IBD and acute GVHD) can significantly activate the immunomodulatory function of MSCs through IFN‐γ and other inflammatory cytokines; upregulate the expression of IDO, PD‐L1, and other immunomodulatory molecules; and enhance the effect of Treg induction [116]. In contrast, chronic fibrotic microenvironments (such as chronic GVHD and advanced OA) lack sufficient inflammatory signals to activate MSCs, and the sustained low‐grade inflammation will lead to Treg instability, resulting in poor long‐term efficacy [117, 118].

#### 4.4.4. Limitations and Translational Challenges of MSC–Treg Axis–Based Therapies in Preclinical and Clinical Settings

Despite the promising preclinical results discussed above, MSC‐based therapies targeting the MSC–Treg axis have encountered significant challenges, with many trials failing to meet their primary endpoints or demonstrating inconsistent efficacy across different disease settings. We use SLE, which has poor clinical translation, as an example to discuss and analyze its limitations.

A randomized, double‐blind, placebo‐controlled trial of allogeneic umbilical cord–derived MSCs for lupus nephritis was abandoned after 18 patients were enrolled, when it became clear that the treatment would not demonstrate a positive effect over standard immunosuppression [119]. Supporting this negative result, a phase I trial in refractory LN reported a complete remission rate of only 33.3% and a partial response rate of 44.4% during the first 3 months postintervention, which declined thereafter. The study concluded that a single dose of AD‐MSCs may not be adequate to maintain long‐term remission [120]. A meta‐analysis of 12 studies further confirmed the low efficacy ceiling; the pooled rate of complete renal remission at 12 months was only 28.1% [121]. One possible reason is that the type I interferon microenvironment in SLE patients markedly impairs immune cells, making it difficult for Tregs to regain normal suppressive function even following MSC‐induced expansion [122].

## 5. Therapeutic Potential and Clinical Considerations of MSCs and MSC‐Derived Exosomes

MSC therapy and MSC‐derived exosome therapy represent two promising strategies for immune modulation and tissue repair, offering distinct mechanisms of action and unique clinical considerations for therapeutic applications. Conventional cellular therapy mainly achieves immunomodulatory effects through the direct administration of MSCs [123]. Clinical studies have shown that MSC infusion is both effective and well‐tolerated in the treatment of diseases such as SLE and CD‐associated strictures [124, 125]. Moreover, standardized MSC infusion protocols have demonstrated favorable short‐term safety profiles, with large‐scale studies reporting relatively low incidences of severe adverse events [126]. Nevertheless, cell‐based therapy still presents potential risks, including immune rejection, tumorigenic potential, and thromboembolic complications [127]. In recent years, MSC‐derived exosomes have attracted considerable attention as an alternative therapeutic strategy. These EVs exhibit biological activities similar to those of their parental MSCs while offering several advantages, including reduced immunogenicity, enhanced stability, convenient storage, and greater potential for standardized manufacturing [128]. As a cell‐free therapeutic modality, MSC‐derived exosomes have shown immunomodulatory effects comparable to, and in some cases superior to, those of MSCs across various disease models. For example, when integrated with gene delivery platforms, MSC‐derived exosomes can promote Treg accumulation and accelerate wound healing in diabetic models [129]. Likewise, in mouse models of OA, they effectively alleviate inflammation without causing significant adverse effects [130]. Compared with MSC‐based cellular therapy, exosome‐based therapy appears to offer superior safety profiles. The therapeutic potential of MSCs and their derived exosomes largely stems from their strong immunomodulatory properties, regenerative capacity, and broad availability.

Their immunoregulatory effects are mainly reflected in their ability to promote Treg proliferation and differentiation via multiple signaling pathways [131], to regulate macrophage polarization [65, 93], and to inhibit proinflammatory Th1 and Th17 responses [43]. Despite these benefits, several challenges remain for clinical translation. For example, within the tumor microenvironment, tumor‐associated MSCs may facilitate Treg expansion, thereby contributing to immune escape and tumor progression [132]. Furthermore, therapeutic outcomes are significantly influenced by factors such as donor heterogeneity, tissue source, and preconditioning strategies. For instance, MSCs cultured under serum‐free conditions exhibit enhanced antifibrotic activity and improved Treg‐inducing capacity [133], whereas dexamethasone pretreatment has been reported to reduce MSC therapeutic efficacy in multiple sclerosis models by decreasing Treg numbers [134].

## 6. Challenges and Future Directions

Tregs may experience functional impairment or undergo conversion into pathogenic Teff lineages in vivo, particularly within inflammatory microenvironments, a process commonly referred to as Treg instability. In such cases, the transition of Tregs into Teffs can initiate excessive proinflammatory responses, ultimately leading to tissue and cellular damage [135]. In addition, Tregs are not functionally uniform. Their heterogeneity gives rise to multiple subpopulations with distinct immunosuppressive capacities, and Tregs derived from different sources or at different differentiation stages may exhibit markedly different therapeutic potentials. To address these challenges, emerging evidence indicates that MSCs and their derived exosomes can enhance Treg stability through various mechanisms while promoting the expansion or induction of functionally superior Treg subsets. MSCs derived from different tissues also exhibit distinct immunomodulatory properties, and variability among MSC sources may contribute to inconsistent therapeutic outcomes and limited reproducibility in experimental studies. For example, adipose‐derived MSCs exhibit notable advantages in tissue repair, whereas umbilical cord–derived MSCs appear to be more effective in promoting regeneration within the reproductive system [136]. In addition to tissue origin, culture conditions (including serum supplementation [137], passage number [138], and cryopreservation [139]) profoundly affect MSCs’ capacity to induce Tregs, yet these variables are often underreported, undermining cross‐study reproducibility. Understanding these functional differences associated with tissue origin may facilitate the selection of optimal MSC sources for specific clinical applications. Although international guidelines, such as MISEV 2023, have provided recommendations for exosome production [140], many aspects of the production process, including isolation, storage, and large‐scale manufacturing, remain underdeveloped. More critically, the field lacks a validated potency assay that predicts in vivo Treg‐inducing activity, and there is no consensus on dosing regimens or release criteria [141, 142]. Consequently, establishing standardized production systems capable of ensuring consistent therapeutic performance for MSCs and their exosomes remains a major challenge. To improve functional efficacy and achieve targeted delivery, various cell engineering strategies have been developed for MSCs and their exosomes. For instance, MSCs engineered to overexpress CEACAM1 following IFN‐γ stimulation can effectively inhibit T cell proliferation and activation [143]. More advanced engineering techniques involve multifaceted modifications of MSCs, such as designing MSC aggregates to enhance immunomodulatory effects [144]. Engineering of exosomes generally follows two main strategies: modifying parental MSCs to produce engineered exosomes or directly altering exosomes to enhance their biological functions [69, 145]. Although many of these approaches have shown encouraging results in preclinical studies, further research is needed to assess their feasibility and effectiveness in clinical settings. Overall, the development of engineered MSCs and MSC‐derived exosomes represents an important direction for future investigation.

## 7. Conclusion

MSCs regulate Tregs through multiple mechanisms that promote their induction, expansion, and functional stability. These mechanisms include the secretion of soluble mediators (e.g., TGF‐β, IDO, PGE2, IL‐10, and HGF), cell‐contact–dependent signaling pathways such as PD‐L1/PD‐1 and Notch signaling, exosome‐mediated transfer of bioactive molecules, mitochondrial transfer, and metabolic and epigenetic regulation. Through these coordinated pathways, the MSC–Treg axis plays a crucial role in maintaining immune homeostasis and shows considerable therapeutic potential in various inflammatory and immune‐mediated diseases. In addition, MSC‐derived exosomes, as cell‐free therapeutic agents with improved safety and stability, represent a promising alternative strategy for immunomodulatory therapy. Nevertheless, challenges such as cellular heterogeneity and the limited translational validation of preclinical findings remain. Future translational and clinical studies are therefore essential to fully realize the therapeutic potential of MSC‐ and exosome‐based interventions.

## Author Contributions

**Meng Yuan and Ting Sun**: writing – original draft, formal analysis. **Peter Muro and Yaru Qiao**: review and editing, funding acquisition. **Bo Wang**: writing – review and editing, software. **Fei Mao and Xiangjun Zhou**: conceptualization, writing – review and editing, funding acquisition.

## Funding

This study was funded by the Key Project of the Health Commission of Jiangsu Province (Grant K2024015).

## Disclosure

All authors have critically reviewed drafts of the manuscript and approved the final version.

## Ethics Statement

The authors have nothing to report.

## Consent

The authors have nothing to report.

## Conflicts of Interest

The authors declare no conflicts of interest.

## Data Availability

Data sharing is not applicable to this article, as no datasets were generated or analyzed during the current study.
